# Evaluating functional and health outcomes for patients with spina bifida 5 and 10 years post-transition into adult care

**DOI:** 10.1186/1743-8454-7-S1-S27

**Published:** 2010-12-15

**Authors:** Irene Chan, Elysia Adams, Beverly Irwin, Paul Thiessen, Bonnie Sawatzky

**Affiliations:** 1 Department of Pediatrics, British Columbia Children’s Hospital, 4480 Oak Street, Vancouver, B.C.Canada

## Background

In BC, pediatric patients with spina bifida are followed by a multidisciplinary clinic at BC Children’s Hospital. Upon graduation, patients transition into the adult healthcare system where they must coordinate their own care. The purpose of this study is to determine the functional and health outcome of graduates transitioning from the spina bifida clinic at BC Children’s Hospital.

## Materials and methods

Graduates of up to 10 years (1999-2008) from the pediatric spina bifida clinic were mailed a questionnaire. It consisted of 11 sections, including two standardized questionnaires on quality of life (QOL), the Medical Outcomes Study 36-item short-form (SF-36) and Spina Bifida-specific Health Related QOL (SBHRQOL).

## Results

113 graduates were identified; 19 were lost to follow-up. 27 (29%) questionnaires were returned. 96% graduated from high school; 63% went on to post-secondary education. 30% had never been employed. 85% lived with parents. 48% had never been in a relationship. 81% were satisfied with their present bowel care; 38% were always clean and 15% had no bowel control. 85% were satisfied with their present bladder care; 22% were always dry and 11% had no bladder control. 19% voided independently and 78% self-catheterized. The wheelchair (58%) was most commonly used for community ambulation. 74% drove or utilized public transit. 93% were satisfied with life in general. The SF-36 physical function domain had the lowest average score (56/100). SBHRQOL mean score was 191. Overall, 89% were satisfied with their ambulation. 96% were satisfied with the care from the pediatric clinic; 52% were satisfied with their adult care. 96% of patients had a family physician; of these, 61% felt that their physician understood spina bifida.

## Conclusions

Health and functional outcomes may indicate the level of independence of adults living with spina bifida. Physical and financial dependence may limit these graduates from full independence. Most patients completed high school; fewer pursued higher education. Many patients demonstrated independence in the community by driving or using transit. Although largely satisfied with their bladder and bowel function, patients continued to have episodes of incontinence. Bladder function was mostly managed by self-catheterization.Overall, the majority of patients were satisfied with life in general, however, our results suggest that more physical support is required for adult graduates. Our results show that fewer adults were satisfied with adult care than in the pediatric clinic; this may result from a perception that their adult healthcare providers inadequately understood spina bifida.

